# IL-1 receptor antagonist restores IL-18 NK cell axis in systemic JIA

**DOI:** 10.1186/1479-5876-10-S3-P45

**Published:** 2012-11-28

**Authors:** Sebastiaan J Vastert, Wilco De Jager, Bo Jan Noordman, Berent J Prakken, Nico M Wulffraat

**Affiliations:** 1 Dept. of Pediatric Immunology, University Medical Center Utrecht, Utrecht, the Netherlands

## Background

Systemic onset juvenile idiopathic arthritis (SoJIA) is an acquired auto-inflammatory disease characterized by systemic inflammation and innate immune activation reflected by uncontrolled production of cytokines such as IL-1, IL- 6 and IL-18. In SoJIA, NK cell function is severely hampered despite high levels of IL-18.

We recently found that defective phosphorylation of the IL-18 receptor beta is responsible for the deficient IL-18-NK cell axis in SoJIA.

## Aim

To study first line treatment with recombinant IL-1 receptor antagonist (rIL-1RA, Anakinra) in 16 newly diagnosed and steroid naïve systemic onset JIA patients.

## Materials and methods

Clinical outcome was measured using ACRp70 and ACRp90. Furthermore, NK cell lytic function, inflammasome activity and cytokine levels in plasma were assessed during follow up (max 3 years).

## Results

Here we show that patients with SoJIA have increased inflammasome activation leading to elevated IL-18 levels. First line treatment in steroid naïve patients, with rIL-1RA effectively down-regulated IL-18 levels through suppression of inflammasome activation and led to rapid resolution of clinical features in 87% (ACRp90) of patients. Furthermore, using rIL-1RA as first line treatment approach the defective IL-18-NK cell axis is restored as shown by improved lytic NK cell function and regaining of the NK cell responsiveness to IL-18 stimulation.

## Conclusions

These data suggest that the mechanisms of inflammatory control induced by rIL-1RA in SoJIA patients involves more than blocking IL-1R. Our data show that rIL-1RA directly targets the inflammasome and restores the IL-18 NK cell axis as well.

